# The Effect of Various Fillers on the Properties of Methyl Vinyl Silicone Rubber

**DOI:** 10.3390/polym15061584

**Published:** 2023-03-22

**Authors:** Yun Chen, Kun Wang, Chong Zhang, Wei Yang, Bo Qiao, Li Yin

**Affiliations:** Beijing Institute of Smart Energy, Beijing 102299, China; chenyunsgri@163.com (Y.C.);

**Keywords:** silicone rubber, filler, mechanical properties, breakdown strength, hydrophobicity

## Abstract

Silicone rubber (SIR) has been widely used in electrical insulation fields, and the introduction of new materials is very important for the performance improvement of SIR composites. In this work, four different fillers, including aluminium hydroxide (ATH), yimonite (YMT), boron nitride (BN) and mica-filled SIR composites were prepared, and the vulcanization behavior, mechanical properties, insulation performance and hydrophobicity of the SIR composites were investigated and compared. Both BN- and mica-filled SIR composites showed excellent insulation performance, while the ATH-filled SIR composite exhibited the best mechanical properties with an elongation at break of 230% and a tensile strength of 2.9 MPa. The SIR/BN composite showed a breakdown strength of 29.2 kV/mm with a 5% failure rate. The addition of YMT deteriorated the insulation performance of SIR but improved the elongation at break and hydrophobicity, with an elongation at break increasing from 115% to 410% and the static contact angle improving from 109.8° to 115.6°.

## 1. Introduction

Silicone rubber (SIR) has been widely used in high-voltage composite insulators or electronic packaging owing to its good electrical performance, including tracking and erosion resistance, breakdown strength and dielectric properties [1,2,3]. The excellent hydrophobicity of SIR can reduce the possibility to accumulate stain or moisture to prevent the generation of leak current. SIR materials comprise reinforcing fillers for the desired mechanical properties and extending fillers to improve the electrical properties. Fillers, such as aluminium hydroxide (ATH), fume, precipitated and quartz silica, barium titanate, alumina and calcium carbonate, were used for these purposes [4,5,6,7]. The type, shape, size, content and surface treatment conditions of the fillers are of great importance and affect the electrical properties of SIR.

The mechanical properties of SIR itself are poor, and the reinforcing-filling system is currently the most commonly used for rubber, which generally includes two categories: one is reinforcing fillers, such as carbon black and silica [8]; the other is inactive fillers, such as mica powder, talcum powder, etc., whose role is mainly to give products acid and alkali resistance, heat resistance, chemical corrosion resistance and other properties, and can reduce costs. In addition to the reinforcing filler silica, the filler commonly used in SIR composite insulators also has ATH for the purpose of improving the insulation performance and heat resistance of the insulator [9,10]. Among the fillers, the effect of ATH and silica fillers with different types, sizes and concentrations are extensively investigated in laboratory studies [11,12,13,14]. Samuel Ansorge et al. [15] found that the SIR composite material with ATH had better performance than that without ATH. Composites filled with hydrophobically-modified ATH possessed the best tracking resistance. In the commercial composite insulator umbrella skirt sheath material, the content of ATH filler generally varies from 100 phr to 120 phr. The SIR composite with a low content of ATH exhibits a decreased flame retardance, while a high content of ATH has a negative effect on hydrophobicity. The content of silica should be 30~40 phr. which ensures the SIR composite with good mechanical properties, hydrophobicity and hydrophobicity migration, and also reduces costs.

As far as the current preparation system of SIR composite insulators is concerned, the status of silica and ATH is unshakable, and only the amount of ATH added is sufficient to meet the operation of composite insulators in normal environments [16]. However, there are still some problems to be solved in the composite insulators prepared by the current system, and few studies have been conducted on the use of new materials to fill SIR, and the influence of new materials on the properties of SIR is very important for the performance improvement of SIR composites [17,18,19].

Boron nitride (BN) itself has high thermal conductivity, which can effectively improve the thermal conductivity of composite materials when applied to rubbers, and the use of boron nitride-filled SIR can improve the thermal conductivity of SIR [20,21,22,23]. BN is also an insulating material [24], and its overall performance is similar to that of traditional filler ATH. Mica is a natural mineral composed of a layered silicate structure with excellent insulation performance [25,26,27], which has played a role as the reinforcement filler in different types of polymers. Yimonite (YMT), also known as illite/smectite mixing layer mineral clay, is a transition mineral transforming from smectite to illite [28], which has been found in south-east China in recent years. The excellent reinforcing effect of the clay has been well demonstrated [29,30,31,32], but the investigation on YMT is very few. In this work, four different fillers, including ATH, YMT, BN and mica were mechanically incorporated into SIR matrix, and the mechanical properties, insulation performance and hydrophobicity of the SIR composites were investigated and compared. The possibility of their application in the composite insulation field was discussed to provide more fillers as options for the preparation of composite insulators.

## 2. Materials and Methods

### 2.1. Materials

Methyl vinyl silicone rubber (SIR), vinyl content 0.08%, molecular weight 670,000, was obtained from Zhejiang Xin’an Chemical Group Co., Ltd., Hangzhou, China. ATH (2~5 µm, 97.0%) was bought from Zhongke Flame-retardant New Material Co., Ltd., Hefei, China. BN (5~10 μm, 99.0%) was Yinuo Material Co., Ltd., Qinhuangdao, China. YMT (~1.75 μm) was provided by Zhongkenada Co., Ltd., Shangsi, China. Mica (97%) was provided by Jinghang Mineral Products Co. Ltd., Lingshou, China. 2,5-Dimethyl-2,5-di(tert-butyl peroxy)hexane (DBPH) (Trigonox 101) was purchased from TNJ Chemical Industry Co., Ltd., Hefei, China. All the above-mentioned materials were used as received.

### 2.2. Preparation

The experimental formula of composite materials is shown in Table 1. Firstly, the SIR was plasticized in the two-roll mill, and then the vulcanizing agent DBPH was added and mixed for 5 min. The mixture was taken out as a master glue to ensure that the vulcanizing agent content of each group of composite materials was consistent, and then divided into five equal parts, one of which was pure SIR, and the remaining four parts were separately added by ATH, YMT, BN and mica. The optimal vulcanization time (t_90_) was determined using the rotorless vulcanization instrument (Shanghai Dejie Machinery Equipment Co., Ltd., Shanghai, China) and then the composites were vulcanized using a hot-pressing vulcanization machine (vulcanization conditions: 170 °C × t_90_).

### 2.3. Characterization and Measurement

The shape of the vulcanization curve is related to the temperature of the test and the characteristics of the rubber material, and the MDR-2000 computerized rotorless vulcanizer was used to measure the vulcanization characteristics of SIR composites, and the preparation system of the composite materials is correct according to the obtained vulcanization curve, and the positive vulcanization time is obtained to prepare the vulcanized rubber.

GT-TC2000 tensile machine is adopted to test the mechanical properties of composite materials with a tensile speed of 500 mm/min according to the requirements of the national standard.

The crosslinking density of composites was tested using the equilibrium swelling method. The vulcanized rubber was soaked in toluene for 72 h at room temperature, and the mass of the sample before and after soaking was recorded, calculated by the formula [33]:

(1) $$v_{e}=\frac{1}{2}\left[\frac{\ln\left(1-v_{2}\right)+v_{2}+\chi v_{2}^{2}}{v\cdot v_{2}^{\frac{1}{3}}}\right]$$

where *v_e_* is the crosslinking density of the composite; *v_2_* is the swelling volume rate of rubber (excluding filler), *χ* is the interaction parameter between the rubber phase and solvent phase (SIR and toluene interaction parameter is 0.465) [34] and *v* is the molar volume of solvent. In addition, *v_2_* was calculated according to Equations (2)–(4):

(2) $$v_{2}=v_{1}/\left(v_{1}+v_{sol}\right)$$

(3) $$v_{sol}=\left(M_{2}-M_{1}\right)/\rho_{sol}$$

(4) $$v_{1}=M_{3}/\rho$$

where *v_sol_* is the volume of solvent absorbed by the rubber after swelling; *v*_1_ is the volume of the rubber phase (excluding filler); *ρ_sol_* is the density of the solvent; *ρ* is the density of rubber without filler; *M*_1_ is the mass of the composite before swelling; *M*_2_ is the mass of the composite after swelling and *M*_3_ is the mass of the rubber phase (without filler).

The breakdown strength of the composites was tested according to the test standard of GB/T1695-2005 using a voltage breakdown tester (HCDJC-50kV, Huace Testing Instrument Co., Ltd., Beijing, China) with a stepping voltage of 1 kV/s. The Weibull probability distribution model is one of the main models to describe the insulation failure distribution of electrical equipment, and the value of its shape parameters can reflect the difference in failure mechanism to a certain extent. By analyzing the electrical aging test data of insulating materials, the International Electrotechnical Commission concluded that the electrical aging life of solid insulation materials follows the Weibull distribution. In this paper, the insulation properties of SIR composites are mainly evaluated by using the classical failure model. The Weibull classical failure model is known as the two-parameter Weibull failure model, which is mainly used to describe problems such as electrical insulation and the breakdown life of electrical equipment. Its failure distribution function is:

(5) $$F\left(x\right)=1-e^{-{\left(x/\alpha \right)}^{\beta }}$$

Its probability density function is:

(6) $$f\left(x\right)=\frac{\beta }{\alpha }{\left(\frac{x}{\alpha }\right)}^{\beta -1}e^{-{\left(\frac{x}{\alpha }\right)}^{\beta }}$$

The reliability function is:

(7) $$R\left(x\right)=1-F\left(x\right)=e^{-{\left(x/\alpha \right)}^{\beta }}$$

Thereinto, $f\left(x\right)\geq 0,1\beta >0,\alpha >0,x$ for testing. In this case, *x* is the breakdown strength. The parameter *β* is a shape parameter and *α* is a scale parameter. The shape parameter *β* can be seen as the slope of the probability curve, and the shape parameter is only a pure value, dimensionless. The change in the scale parameter *α* of the Weibull distribution has the same effect as the change in the scale of the abscissa.

For ease of calculation, take the logarithmic form for Equation (5) to get:

(8) $$\mathrm{lg}\left(-\ln\left(1-F\right)\right)=\beta \left(\mathrm{lg}\alpha -\mathrm{lg}x\right)$$

In addition, *F(x)* can be used by the formula:

(9) $$F\left(x\right)=\frac{i-0.5}{n+0.25}$$

to calculate, where *i* is the number of measurements obtained by the *x* value (breakdown field strength) in ascending order and *n* is the total number of tests for each sample. In this paper, all composites are tested 12 times, so there is *n* = 12.

Finally, the least squares method is used to calculate the shape parameter β and scale parameter α of the Weibull distribution function for Equation (8), so as to obtain the two-parameter Weibull failure model.

The static contact angle was measured using a contact angle measuring instrument. The moisture on the surface naturally volatilizes, which was then measured after 24 h.

The contact angle of the water droplet on a flat surface of the sample was measured using a contact angle system (JC2000D3, Zhongchen Digital Technology Co., Ltd., Shanghai, China). Before measuring, the samples would be wiped with ethanol and ultrapure water in turn to minimize contamination. When a water droplet (~5 μL) was dropped onto the sample, the image of the water droplet was captured by the equipped camera and was then analyzed to obtain the contact angle. At least twelve parallel measurements were perfumed to obtain the average values.

## 3. Results and Discussion

### 3.1. Vulcanization Behavior

**Figure 1:**
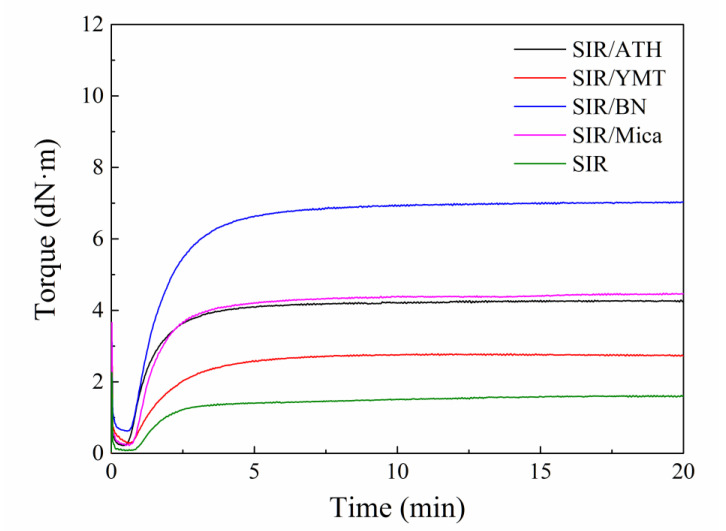
Vulcanization curve of SIR composite with different fillers.

Figure 1 shows the vulcanization characteristic curve of the SIR composite with various fillers. There are three typical zones for vulcanization. The first one is the flat zone, which starts immediately under pressure and heat, showing the lowest torque. The second zone comes right after the first zone, with torque increasing over time, indicating the occurrence of vulcanization, and the curing rate could be determined using the slope of the torque–time curve in this zone. In the last zone, the torque remains fairly constant, demonstrating a fully developed crosslinked structure. The difference between the maximum and minimum torques of the vulcanization curve can characterize the crosslinking density of the composite. It can be seen from the figure that the scorching time and optimal vulcanization time of the composites are almost the same, while the maximum torque and minimum torque vary significantly. Among them, the difference between the maximum torque and the minimum torque of the SIR/BN composite is significantly greater than that of the other four composites. Moreover, the pure SIR is fully cured after 3 min, while the fillers-filled SIR composites are fully cured after 5 min. On the one hand, the fillers could restrict the motion of rubber macromolecules. On the other hand, the reactions between rubber macromolecules and vulcanizing agent molecules might be isolated by the fillers. Therefore, the fillers-filled SIR composites demonstrate a longer vulcanization time than pure SIR.

### 3.2. Mechanical Properties

Table 2 shows the mechanical properties of the SIR composites with different fillers. As can be seen from the table, the hardness of the composite materials increases after adding fillers, and the hardness of the composite materials with BN filler increases the most, increasing from 23 to 62. After adding ATH, the tensile strength of the composite material is the highest, reaching 2.9 MPa, which is ~6.6 times higher than that of the pure SIR, and the elongation at break is also improved, reaching 230%. The elongation at break of the composites is greatly improved after the addition of YMT filler, increasing by ~257% as compared to that of the pure SIR, while the tensile strength only increases from 0.38 MPa to 0.92 MPa. With the addition of BN, the elastic modulus of the composite is the highest, showing a stress at 100% strain of 2.1 MPa, which is more than 500% higher than that of the pure SIR, but the elongation at break does not increase much. For the composite with mica filler, the improvement of elastic modulus is lower than that of the SIR/BN composite, and the elongation at break was not improved. The different mechanical properties among the SIR composites might be due to the different filler shapes, filler–rubber interactions and filler dispersions. The near-linear stress–strain curves are observed for the SIR/ATH composite due to the sphere shape of ATH. The composites filled with the fillers with high aspect ratio, including BN and mica show very high modulus due to the strong filler–filler network at low strain. However, for the YMT-filled SIR composite, the poor filler dispersion has a negative effect on the modulus. When the stretching strain becomes larger, the rubber macromolecules are easily to slip from the surface of the fillers with high aspect ratio, so the stress of the SIR composites filled with BN, mica or YMT increases very little with strain.

**Figure 2:**
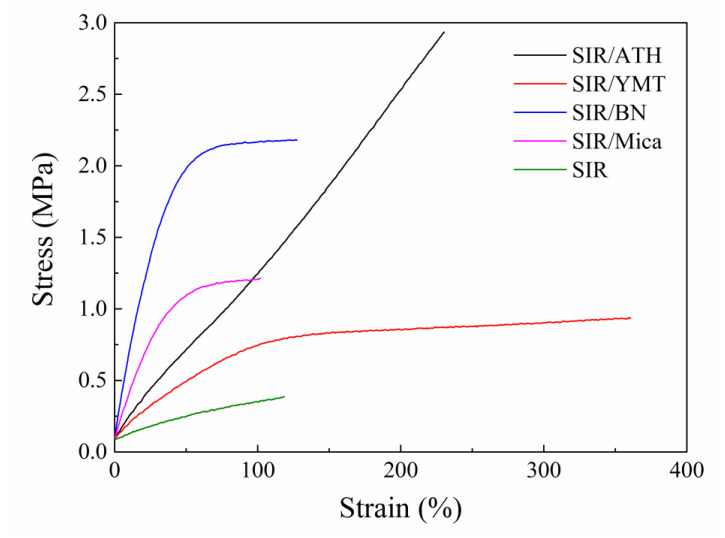
Stress–strain curves of SIR composites with various fillers.

Figure 2 shows the stress–strain curves of the SIR composites with various fillers. As can be seen from the figure, the elastic modulus of the SIR/BN composite material is very large when the elongation is below 50%. However, when the elongation is higher than 50%, the elastic modulus drops sharply, and the tensile strength between 50% and 128% is almost unchanged. This is because BN particles form a strong filler network resulting from the high aspect ratio of the filler [22], and the filler network has a strong force, but the action distance is shorter, in the stretching process the filler network will precede the polymer–polymer network and polymer–filler network fracture, for the composite material with BN, the filler and the polymer do not form an effective network. Therefore, when the filler–filler network is broken, the elastic modulus of the composite material will be greatly reduced, and the subsequent tensile mainly depends on the polymer–polymer network, but the polymer–polymer network strength of SIR is low so that in the subsequent stretching process, the tensile strength is almost not increased. A similar phenomenon is also observed for the composite filled with mica filler.

### 3.3. Crosslinking Density

**Figure 3:**
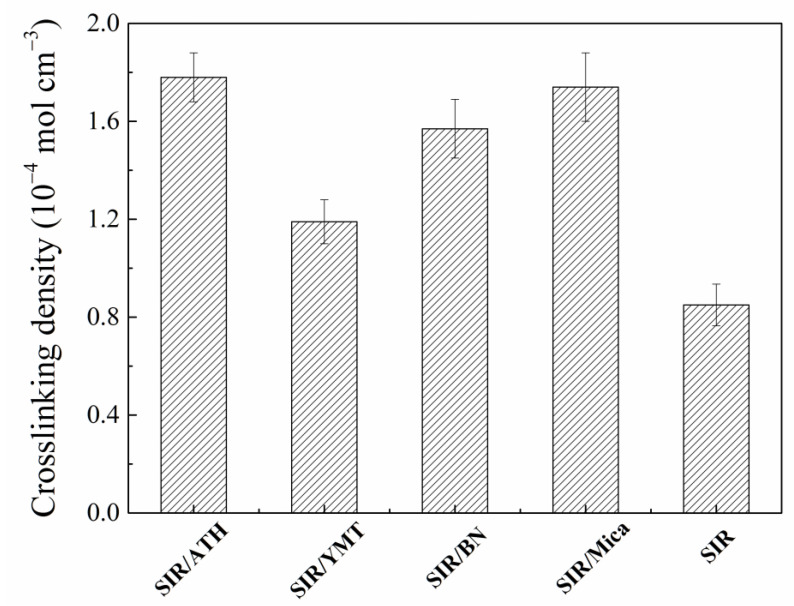
Crosslinking density of SIR composites with various fillers.

Figure 3 shows the crosslinking density of the SIR composites filled with various fillers. It can be seen from the figure that the addition of various fillers has different effects on the crosslinking density of composite materials, among which pure SIR has the lowest crosslinking density. The interactions between filler particles and rubber matrix also contribute to the crosslinking density [32,35], but no filler–rubber interactions in the pure SIR. After the addition of ATH, BN or Mica, the crosslinking density of the composite is nearly twice that of pure SIR, because after the addition of filler, a filler-matrix and filler–filler crosslinking network will be formed inside the composites. As a result, the mechanical properties of the composite material are improved to a certain extent. As for the SIR/YMT composite, the improvement in crosslinking density is much lower than that of the other three SIR composites. On the one hand, there exists plenty of silanol on the YMT surface, which might capture the free radicals produced by the vulcanization agent. On the other hand, the strong interionic interactions between the YMT layers prevent the dispersion of YMT layers in the rubber matrix, resulting in poor rubber–filler interactions.

### 3.4. Breakdown Strength

**Figure 4:**
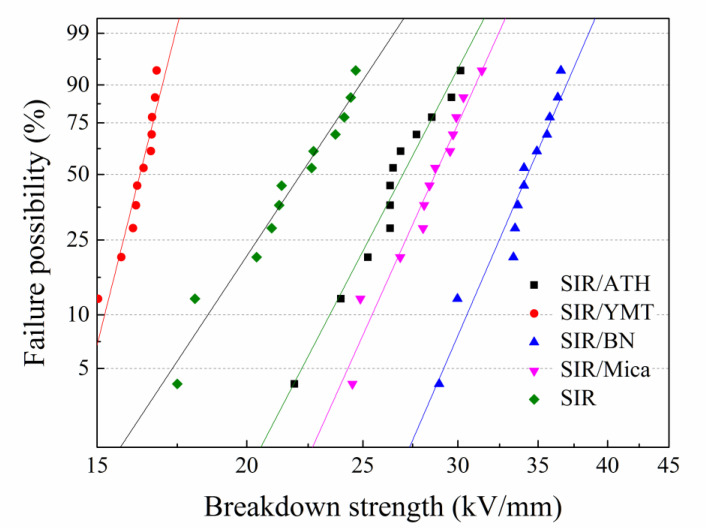
Weibull distribution of SIR composites with various fillers.

Figure 4 shows the Weibull probability distribution of breakdown strength for the SIR composites with various fillers. It can be seen from the figure that for the pure SIR, there is only a 5% probability of electrical breakdown at an electric field strength of 17.5 kV/mm, and most of them occur at an electric field strength of 20–22 kV/mm. This shows that SIR itself has good insulation properties, so SIR will be used as a matrix material for composite insulators in high-voltage environments. With the addition of YMT, the insulation performance of the composite material is worse, because YMT is a natural mineral with a complex chemical composition and a small number of metal cations in the clay layers. Therefore, after being added into the SIR, the presence of these small amounts of metal cations will reduce the insulation performance of the composite material and reduce the breakdown strength. After the addition of ATH, mica or BN, the breakdown strength of the composites is improved. By incorporating the traditional thermally conductive insulation ATH filler, the SIR/ATH composite possesses a breakdown strength of 22.2 kV/mm with a failure rate of 5%, which exhibits a higher insulation performance than the pure SIR, but the improvement on the insulation performance of the composite is still not as good as that of the composites filled with mica or BN fillers. The insulation property of the SIR composite filled with mica filler are between ATH and BN, and the breakdown strength with a failure rate of 5% is 24.3 kV/mm. The insulation performance of the composite with BN is the best among the four fillers, the SIR/BN composite shows a 5% probability of electrical breakdown under the electric field strength of 29.2 kV/mm, which is 67% higher than the initial breakdown strength of the pure SIR, and the electric field strength with a failure rate of 50% is 34.3 kV/mm, which is a very significant improvement on insulation performance.

### 3.5. Static Contact Angle

**Figure 5:**
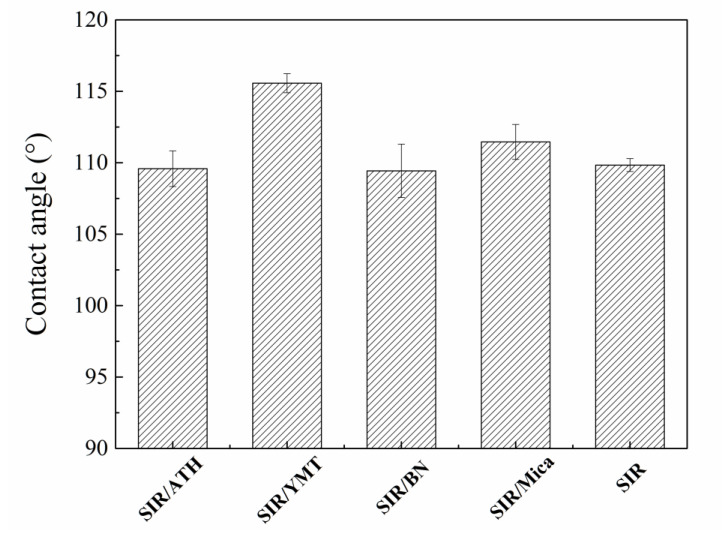
Static contact angle of SIR composites with various fillers.

The hydrophobic characteristic is a key factor of silicone rubber insulators to ensure their safe and stable operation [36]. In contaminated and humid conditions, water molecules gather as independent droplets rather than a continuous water film; thus, the surface maintains a high level of anti-flashover performance. Therefore, the hydrophobic surface of silicone rubber composite is of great significance to prevent the failure of insulators. Figure 5 shows the static contact angle of a composite filled with various fillers. It can be seen from the figure that after adding ATH, BN and mica filler, the static contact angle of the composite material is almost the same as that of the SIR without filler, and it can be considered that the addition of these three fillers has little effect on the static contact angle of the composite material. For comparison, the static contact angle of the SIR composite filled with YMT improves from 109.8° to 115.6°. By incorporating YMT, the hydrophobicity of the SIR composite improves but the breakdown strength decreases, which is very important for insulation materials. The insulation performance may be improved by blocking cations of YMT, which needs further investigation in the future. Moreover, the improvement in mechanical properties by adding YMT is not as good as that by adding ATH, BN or mica. Therefore, we consider the YMT might not be a good candidate filler for insulation materials.

## 4. Conclusions

In this paper, the effects of fillers, including ATH, YMT, BN and mica on the mechanical properties, insulation properties and static contact angle of composites were investigated and discussed. Among them, the composite filled with ATH exhibited a good improvement in mechanical properties and insulation properties, and its mechanical properties were the best among the four SIR composites, with an elongation at break of 230% and a tensile strength of 2.9 MPa. The SIR composite filled with YMT showed the highest elongation at break, reaching 410%, which is much higher than the other SIR composites. Moreover, the addition of YMT would reduce the insulation performance but increase the static contact angle from 109.8° to 115.6°. The composite filled with BN possessed the best insulation performance, the breakdown strength of the composite with a failure rate of 5% is 29.2 kV/mm, much higher than that of the pure SIR, which makes the composite material better adapt to the high-voltage environment and reduce the failure rate of the composite insulator. However, BN has less reinforcing effect on SIR, only improves the elastic modulus of the composite, and the elongation at break is almost not increased. The SIR composite filled with mica filler showed a better insulation performance than that filled with ATH, but the reinforcement effect and the improvement of insulation performance are not as good as BN.

## Figures and Tables

**Table 1:** Experimental formulation of SIR composites with various fillers (unit: phr, per hundred rubber).

Ingredients	Sample Codes				
	SIR/ATH	SIR/YMT	SIR/BN	SIR/Mica	SIR
SIR	100	100	100	100	100
ATH	100	0	0	0	0
YMT	0	100	0	0	0
BN	0	0	100	0	0
Mica	0	0	0	100	0
DBPH	0.5	0.5	0.5	0.5	0.5

**Table 2:** Mechanical properties of SIR composites with various fillers.

Properties	SIR/ATH	SIR/YMT	SIR/BN	SIR/Mica	SIR
Hardness	48	34	62	52	23
Tensile strength/MPa	2.9	0.92	2.1	1.2	0.38
Elongation at break/%	230	410	128	102	115
Stress at 100% strain/MPa	1.2	0.74	2.1	1.2	0.35

## Data Availability

The data presented in this study are available on request from the corresponding author.
